# Effects of blocking developmental cell death on sexually dimorphic calbindin cell groups in the preoptic area and bed nucleus of the stria terminalis

**DOI:** 10.1186/2042-6410-3-5

**Published:** 2012-02-15

**Authors:** Richard F Gilmore, Megan M Varnum, Nancy G Forger

**Affiliations:** 1 Department of Psychology and Center for Neuroendocrine Studies, University of Massachusetts, Amherst, MA 01003, USA

**Keywords:** Bax, bed nucleus of the stria terminalis, calbindin, cell death, preoptic area, sex difference

## Abstract

**Background:**

Calbindin-D28 has been used as a marker for the sexually dimorphic nucleus of the preoptic area (SDN-POA). Males have a distinct cluster of calbindin-immunoreactive (ir) cells in the medial preoptic area (CALB-SDN) that is reduced or absent in females. However, it is not clear whether the sex difference is due to the absolute number of calbindin-ir cells or to cell position (that is, spread), and the cellular mechanisms underlying the sex difference are not known. We examined the number of cells in the CALB-SDN and surrounding regions of C57Bl/6 mice and used mice lacking the pro-death gene, *Bax*, to test the hypothesis that observed sex differences are due to cell death.

**Methods:**

Experiment 1 compared the number of cells in the CALB-SDN and surrounding regions in adult males, females, and females injected with estradiol benzoate on the day of birth. In experiment 2, cell number in the CALB-SDN and adjacent regions were compared in wild-type and *Bax *knockout mice of both sexes. In addition, calbindin-ir cells were quantified within the principal nucleus of the bed nucleus of the stria terminalis (BNSTp), a nearby region that is larger in males due to *Bax*-dependent cell death.

**Results:**

Males had more cells in the CALB-SDN as well as in surrounding regions than did females, and estradiol treatment of females at birth masculinized both measures. *Bax *deletion had no effect on cell number in the CALB-SDN or surrounding regions but increased calbindin-ir cell number in the BNSTp.

**Conclusions:**

The sex difference in the CALB-SDN of mice results from an estrogen-dependent difference in cell number with no evidence found for greater spread of cells in females. Blocking *Bax*-dependent cell death does not prevent sex differences in calbindin-ir cell number in the BNST or CALB-SDN but increases calbindin-ir cell number in the BNSTp of both sexes.

## Background

The sexually dimorphic nucleus of the preoptic area (SDN-POA) was discovered over 30 years ago in rats and is arguably the best-studied sex difference in the mammalian brain [1,2]. Based on measurements in Nissl-stained sections the nucleus is several times larger in volume in male rats than in females, and similar sex differences have been described in the medial POA of other mammals, including gerbils, guinea pigs, sheep, ferrets, hyenas, monkeys, and humans [3-10].

The sex difference in SDN-POA volume in rats depends on estrogenic metabolites of testosterone and is thought to be due to differential cell death during postnatal development. Male rats have more cells in the SDN-POA than do females, beginning by the second postnatal week [11-14], and females have a higher rate of cell death between postnatal days 7 and 10 [12,15,16]. Treating females with testosterone or an estrogen around the time of birth reduces postnatal apoptosis in the SDN-POA [12,14,15,17] and masculinizes SDN-POA volume in adulthood [1,18,19].

Cell death in the developing brain is crucially regulated by proteins of the B cell lymphoma 2 (Bcl-2) family [20]. Bcl-2 itself is antiapoptotic, whereas Bcl-2-associated × protein (Bax) is a proapoptotic family member required for developmental cell death of neurons [21,22]. Female rats have reduced levels of Bcl-2 and higher levels of Bax protein in the SDN-POA during the postnatal cell death period [16]. Moreover, treatment with estradiol reverses the sex difference in the Bcl-2/Bax ratio [23], consistent with the idea that estradiol normally rescues cells in the SDN-POA of males via changes in Bcl-2 family proteins.

Mice overexpressing Bcl-2 or with a targeted deletion of the *Bax *gene have been used to directly examine the role of cell death in the development of neural sex differences [24,25]. Despite its prominence in sexual differentiation research, the SDN-POA was not examined in these studies because mice do not have a discernable SDN-POA based on a Nissl stain [26]. More recently, however, the calcium binding protein calbindin-D28 has been used as a marker of the SDN-POA.

Sickel and McCarthy [27] first showed that male rats have a striking cluster of calbindin-immunoreactive (ir) cells that nearly perfectly overlaps the Nissl-defined SDN-POA in adulthood; this cluster (hereafter referred to as the CALB-SDN) becomes sexually dimorphic by postnatal day 8 under the control of gonadal steroid hormones [27]. Mice also have a calbindin cluster that is exclusive to, or larger in, males [28-31]. As in rats, this sex differences appears to be due to organizational effects of gonadal steroids: gonadectomy of male mice and treatment of females with testosterone on the day of birth reverses the sex difference in the CALB-SDN whereas hormone manipulations in adulthood are without effect [31].

Based on these observations, the CALB-SDN seems to be a good proxy for the SDN-POA. Some confusion exists, however, about whether the sex difference is due to a difference in cell number or in cell position. Whereas some studies find more calbindin-ir cells in this region in male rats or mice [28,31,32], others report no sex difference in overall cell number, suggesting instead that cells are either more densely packed within the (much smaller) CALB-SDN of females and/or that females have more cells in regions surrounding the cluster [29,33,34]. Part of the discrepancy may be due to the subjective nature of defining the CALB-SDN, especially in females. The exact location of cell counts is often not clearly defined and published photomicrographs suggest that the various studies may not all refer to the same cell group.

Given the importance of the SDN-POA in sexual differentiation research and the increased reliance on calbindin as a marker of this nucleus, we sought to resolve several questions: first, whether there is a sex difference in cell number and/or spread of calbindin-ir cells in the CALB-SDN and surrounding regions of mice; second, whether cell number and/or spread are affected by neonatal estradiol treatment of females; and third, whether cell death contributes to sexual differentiation of the CALB-SDN. Based on our current understanding of how the Nissl-defined SDN-POA develops in rats, we hypothesized that there is a sex difference in cell number in the CALB-SDN of mice that is due to estrogen-regulated, Bax-dependent cell death. Our findings support two of these three predictions. We found an estradiol-dependent sex difference in calbindin-ir cell number in mice, both within the CALB-SDN and in regions surrounding the cluster. Surprisingly, however, we did not find support for a role of cell death in establishment of the sex difference: *Bax *gene deletion does not affect the number of cells in the CALB-SDN, although it significantly increases calbindin-ir cell number in the nearby principal nucleus of the bed nucleus of the stria terminalis (BNSTp).

## Methods

### Animals and treatments

All procedures were approved by the Institutional Animal Care and Use Committee at the University of Massachusetts, Amherst. Mice were housed under 14:10 light/dark conditions at 22°C and provided with food and water *ad libitum*.

In experiment 1, C57Bl/6 pups from our breeding colony were injected on the day of birth with either 20 μg of estradiol benzoate (EB; Sigma-Aldrich, St. Louis, MO, USA) in 25 μl vehicle (90% peanut oil and 10% dimethylsulfoxide (DMSO)) or the vehicle alone (N = 6 per group). The dose of EB was based on that previously shown to masculinize BNSTp volume and cell number in mice [35]. Mice were killed and their brains collected between postnatal days 55 to 60. Because calbindin-ir cell number in the POA of mice is not influenced by gonadal hormones in adulthood [30], animals remained gonadally intact. Brains were immersion fixed in 5% acrolein (Sigma-Aldrich) in 0.1 M phosphate buffer for 4 h, and then submerged in 30% sucrose. Coronal sections were cut into three series at 30 μm on a freezing microtome and stored at -20°C in cryoprotectant until use. One of the three series was thionin stained and a second was labeled for calbindin immunoreactivity, as described below.

In experiment 2, wild-type (WT; *Bax +/+*) and *Bax *knockout (KO; *Bax -/-*) mice were generated by pairing mice heterozygous for the *Bax *gene deletion on a C57Bl/6 background. Tail tissue was genotyped for *Bax *gene status via PCR using previously established primers [22]. Animals were killed in adulthood (60 to 90 days of age), and their brains were fixed as above. Sections were cut into two series at 30 μm, one of which was labeled for calbindin. Four groups were compared (N = 6 per group): WT males, WT females, *Bax *KO males and *Bax *KO females. Three animals were dropped from grid counts (defined below) due to histological artifacts (folding or tearing of sections) that precluded accurate counts throughout the grid bilaterally; the final number of animals per group is indicated in each figure.

All measurements reported in this study were made on slides coded to conceal the sex and treatment of the animals.

### Immunocytochemistry for calbindin

Free-floating sections were rinsed three times for 5 minutes in Tris-buffered saline (TBS; 0.05 M Tris, 0.9% NaCl, pH 7.6) then incubated in 0.05 M sodium citrate in TBS for 1 h. Sections were rinsed, placed in 0.1 M glycine in TBS for 30 minute and rinsed again before incubating in blocking solution (20% normal goat serum (NGS; Pel-Freez Biologicals, Rogers, AR, USA), 0.3% Triton-X (Labchem, Inc., Pittsburgh, PA, USA) and 1% H2O2 in TBS) for 30 minutes. Sections were then exposed to primary antibody overnight (1:5,000 mouse monoclonal anti-calbindin-D28k antiserum (C9848; clone CB-955; Sigma-Aldrich)) in 2% NGS, and 0.3% Triton-X. Sections were then rinsed and incubated in secondary antiserum (1:250 biotinylated goat anti-mouse IgG, (Vector Laboratories, Burlingame, CA, USA) in TBS with 2% NGS and 0.32% Triton-X) for 1 h, followed by three 10 minute rinses in TBS containing 0.2% Triton-X. Sections were then incubated in avidin-biotin complex in TBS (ABC Elite Kit, Vector Laboratories), rinsed, and incubated in diaminobenzidine (DAB Kit, Vector Laboratories). Sections were mounted onto slides and coverslipped with Permount (Fisher Scientific, Pittsburgh, PA, USA).

### Defining the CALB-SDN

**Figure 1:**
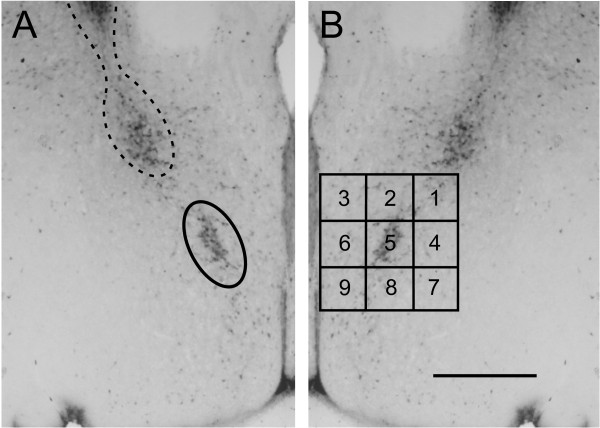
**Ellipse and grid contours designed to quantify calbindin-immunoreactive (ir) cell number in and surrounding the calbindin-ir sexually dimorphic nucleus in the preoptic area (CALB-SDN)**. The photographs in **(A) **and **(B) **are mirror-image duplications of a single image from a male mouse. (A) For CALB-SDN counts, an ellipsoid contour (solid line) was placed as indicated. A second calbindin-ir cell cluster was found dorsolateral to the CALB-SDN. These cells were determined to be the ventral extension of the bed nucleus of the stria terminalis (BNSTp) (dotted line) and were not included in CALB-SDN counts. **(B) **For counts of cells within and surrounding the CALB-SDN, a nine-block grid was placed as shown and counts were made within each block of the grid. Scale bar = 400 μm.

Sections through the medial POA were examined using a video camera connected to a light microscope and analyzed with the aid of StereoInvestigator software (MicroBrightfield, Williston, VT, USA). As reported previously, a distinctive cluster of calbindin-ir cells was found in males. This cluster was ellipsoidal in shape and centered at about 670 μm dorsal to the optic chiasm and 275 μm lateral to the third ventricle; it angled away from the third ventricle dorsolaterally (see Figure 1). It was found in a single immunostained section or in two sections in males; because every third 30 μm section was immunostained, this implies that the cluster spans between 90 to 180 μm rostrocaudally. Depending on the exact plane of sectioning, the cluster was present in sections in which the anterior commissure crossed the midline or, more often, just caudal to this point. Females also had calbindin-ir cells in this region, which either did not form a distinct cluster or formed a very small cluster.

We also noted a second calbindin-ir cell group in both sexes that was more dorsal and lateral to the CALB-SDN described above (Figure 1). Depending on the plane of section, this cell group was either present in the same section as the CALB-SDN, or in the next section caudally. Sparsely distributed calbindin-ir cells were sometimes seen connecting the two cell groups. Perhaps for this reason, some previous studies may have included this more dorsal cell group in CALB-SDN analyses (as suggested by photomicrographs in refs. 29 and 30, for example). However, by comparing the sections immunoreacted for calbindin with adjacent sections stained with thionin, we concluded that the more dorsal cluster comprises the ventral-most portion of the BNSTp. These cells were therefore excluded from the CALB-SDN contour and grid counts described below (unless noted) and were included as part of the BNSTp in experiment 2.

### Calbindin-ir contour and grid counts

Calbindin-ir cells in the POA were quantified in experiments 1 and 2 using two methods: contour counts and grid counts (Figure 1). A contour (defined below) was designed based on the size, shape and location of the CALB-SDN in males, allowing us to count calbindin-ir cells in a consistent location in all animals. A nine-block grid, centered on the CALB-SDN, allowed us to count cells both within the cluster and in regions of the POA surrounding the cluster.

### Contour counts

Based on pilot work, we designed an ellipsoid contour that was superimposed on sections through the POA to count CALB-SDN cells in a consistent manner across all animals. The contour (65,000 μm^2^; major axis 365 μm and minor axis 220 μm) was aligned with respect to the optic chiasm and third ventricle (ventral-most part of the contour approximately 675 μm dorsal to the optic chiasm and center of the contour 275 μm lateral to the third ventricle; see Figure 1A). The size of the contour was somewhat larger than the CALB-SDN found in males so that regardless of individual differences or differences in plane of sectioning the entire cluster would be included in all animals. Because the total number of calbindin-ir cells in this region was relatively small (< 100), sampling was not required; all cells that came into focus within the section and were moderately to darkly labeled were counted directly. Counts were performed on the left and right sides of the relevant sections and the two highest counts were summed for each animal. In the majority of cases, the maximal counts turned out to be the left and right sides of a single section. In the remaining cases (presumably due to asymmetrical sectioning), the two maximal counts came from adjacent sections.

To address the issue of spread of calbindin-ir cells, we also designed a grid (total size 525 μm by 525 μm) divided into nine uniform blocks that was superimposed on the same sections used for contour counts. The grid was aligned with the edge of the third ventricle and positioned so that the large majority of cells in the CALB-SDN as defined above fell within the central block of the grid. The number of calbindin-ir cells was counted within each block of the grid.

### Calbindin-ir cell counts in the BNSTp

The number of calbindin-ir cells was counted in the BNSTp of wild-type and *Bax *KO mice in experiment 2. Because the BNSTp is large and contains thousands of calbindin-ir cells, the direct counting of all cells was not possible. Instead, the optical fractionator function of StereoInvestigator was used for random systematic sampling and determination of total calbindin-ir cell number. A counting frame of 25 μm × 25 μm and a counting grid of 85 μm × 85 μm was used to sample regions throughout the nucleus; counts of medium to darkly labeled cells were made using standard stereological rules, as previously described [36].

### Total cell density in the POA of wild-type and *Bax *KO animals

Coverslips were soaked off after the calbindin-ir cell counts were made in experiment 2, and sections were counterstained with thionin. The ellipsoidal contour was again superimposed on sections containing the CALB-SDN, as described above, and all cells within the contour (calbindin-positive plus thionin only) were counted.

### Statistics

One-way analysis of variance (ANOVA) was used to compare mean calbindin-ir cell number based on ellipsoid contour or grid counts (individual grid blocks and total) among the three groups in experiment 1. Two-way analyses of variance (sex-by-genotype) were used to compare calbindin-ir cell number in the CALB-SDN and BNSTp of WT and *Bax *KO mice in experiment 2. Fisher's least significant difference (LSD) post hoc tests were performed only following significant main effects or interactions.

## Results

### Experiment 1: cell number in the CALB-SDN of males, females, and females treated with EB at birth

#### Contour and grid counts

**Figure 2:**
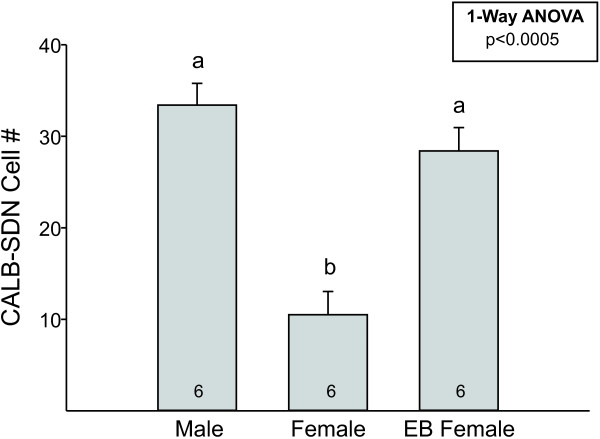
**Mean (± SEM) number of cells in the calbindin-immunoreactive sexually dimorphic nucleus in the preoptic area (CALB-SDN) of males, females, and females treated with a single injection of estradiol benzoate (EB) on the day of birth**. N = 6 per group. Different letters above bars indicate significant differences between groups in *post hoc *tests.

An ellipsoid contour was used to count cells in the CALB-SDN of control males, control females, and females treated with a single injection of EB on the day of birth (Figure 2). There was a main effect of group (F_2, 15 _= 30.0; *P *< 0.0005) with a greater number of CALB-SDN cells in males than in control females (*P *< 0.0005). Females treated with EB on the day of birth also had significantly more cells in the CALB-SDN than control females (*P *< 0.0005) and were not different from males.

**Figure 3:**
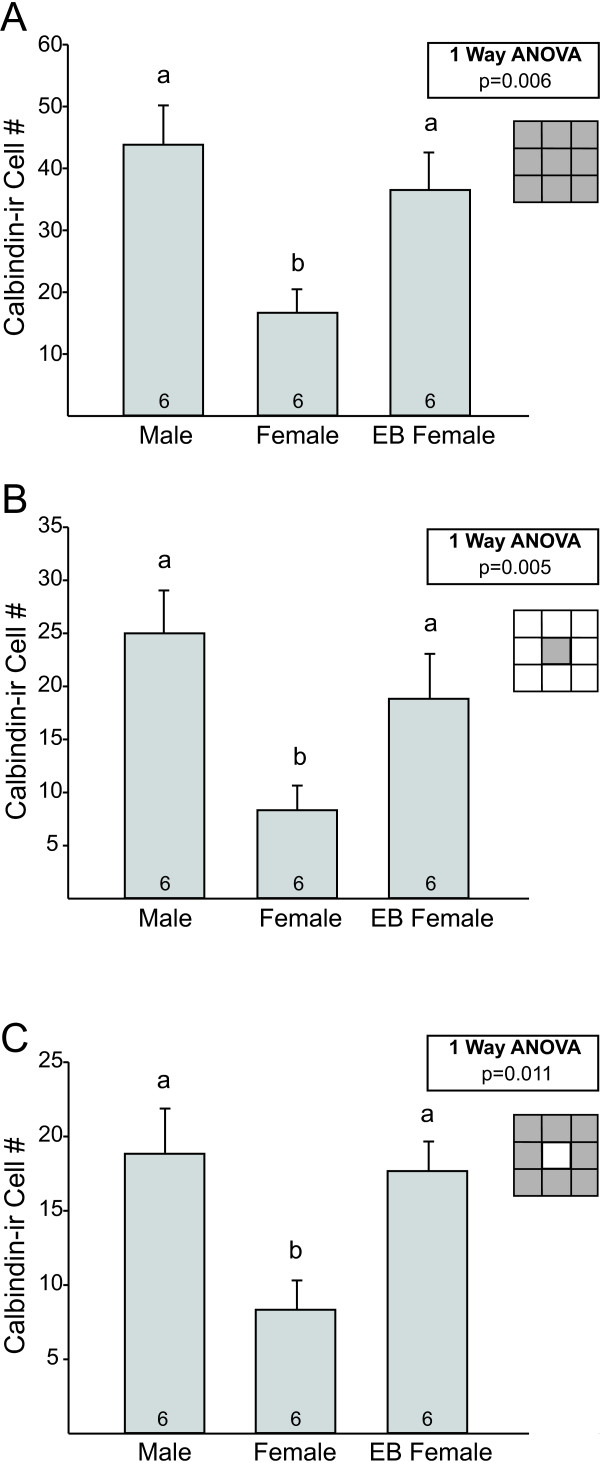
**Grid analysis of calbindin-immunoreactive (ir) cells in and around the calbindin-ir sexually dimorphic nucleus in the preoptic area (CALB-SDN) in males, females, and females treated with a single injection of estradiol benzoate (EB) on the day of birth**. Mean (± SEM) calbindin-ir cell number **(A) **within the total grid (sum of blocks 1 to 9); **(B) **in the central block of the grid only (which captures the majority of the CALB-SDN); and **(C) **in blocks 1 to 4 and 6 to 9 of the grid, reflecting calbindin-ir cells in regions surrounding the CALB-SDN. N = 6 per group. Different letters above bars indicate significant differences between groups in *post hoc *tests.

A grid was used to examine calbindin-ir cells in a larger region centered on and surrounding the CALB-SDN (Figure 3). We found a significant effect of group on calbindin-ir cell number within the entire grid (F_2, 15 _= 7.4 *P *= 0.006). Males and EB-treated females had more calbindin-ir cells than control females (*P *= 0.002 and *P *= 0.016, respectively), and did not differ significantly from each other (Figure 3A). Counts within each of the nine individual blocks of the grid also were compared. The central block (block 5, which included the CALB-SDN as defined above) contained about half of the total calbindin-ir cells in each group and showed significant group differences (F_2, 15 _= 7.6; *P *= 0.005). As expected, males and EB-treated females had significantly more calbindin-ir cells than control females in this block (*P *= 0.002 and *P *= 0.029, respectively; Figure 3B). No other single block showed a significant group difference in calbindin-ir cell counts, but males tended to have more calbindin-ir cells than females in every block. Indeed, when counts from all blocks except the central block were combined (that is, the sum of blocks 1 to 4 and 6 to 9), a significant main effect was observed (F_2, 15 _= 6.1; *P *= 0.011), with more calbindin-ir cells in males (*P *= 0.006) and EB-treated females (*P *= 0.013) than in control females (Figure 3C).

We noted that in some cases a few cells of the CALB-SDN and/or of the ventral-most BNSTp extended into block 1 (that is, the dorsolateral corner of the grid). To determine whether this affected our results, we reanalyzed calbindin-ir counts, omitting all cells in both blocks 1 and 5. The pattern was the same: males and EB females had more calbindin-ir cells than control females (blocks 2 to 4 plus 6 to 9; *P *= 0.026; *P *= 0.029, respectively; data not shown). Thus, both within the CALB-SDN and in the regions immediately surrounding the CALB-SDN, males have more calbindin-ir cells than females.

### Experiment 2: effects of *Bax *gene deletion on calbindin-ir cell number in the CALB-SDN and BNSTp

#### CALB-SDN and grid cell counts

**Figure 4:**
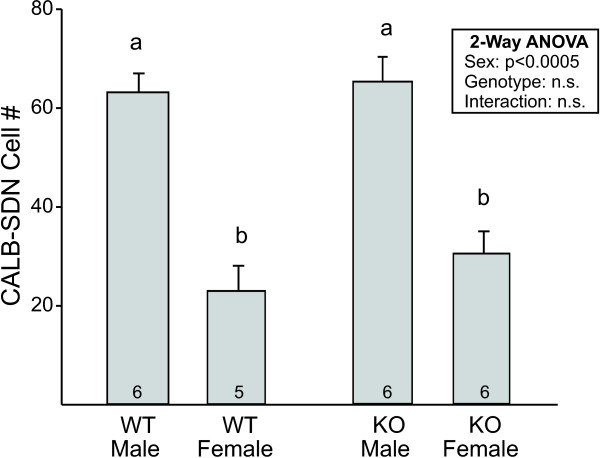
**Mean (± SEM) number of cells in the calbindin-immunoreactive sexually dimorphic nucleus in the preoptic area (CALB-SDN) of wild-type (WT) and *Bax *knockout (KO) males and females**. There was a sex difference, but no effect of *Bax *genotype on CALB-SDN cell number. The number of animals per group is indicated at the base of each bar. Different letters above bars indicate significant differences between groups in *post hoc *tests.

To test whether the sex differences in calbindin-ir cell number observed in experiment 1 were due to cell death, we performed contour and grid counts in adult wild-type and *Bax *knockout mice. Overall immunostaining was darker in this study than in experiment 1, leading to a greater number of medium-to-darkly stained cells across groups. However, the ratio between the sexes remained the same (compare Figure 2 and 4) and two-way ANOVA revealed the expected main effect of sex on cell number in the CALB-SDN (Figure 4; F_1, 20 _= 65.5; *P *< 0.0005). There was no significant effect of *Bax *genotype or sex-by-genotype interaction on this measure (both *P *values > 0.3).

**Figure 5:**
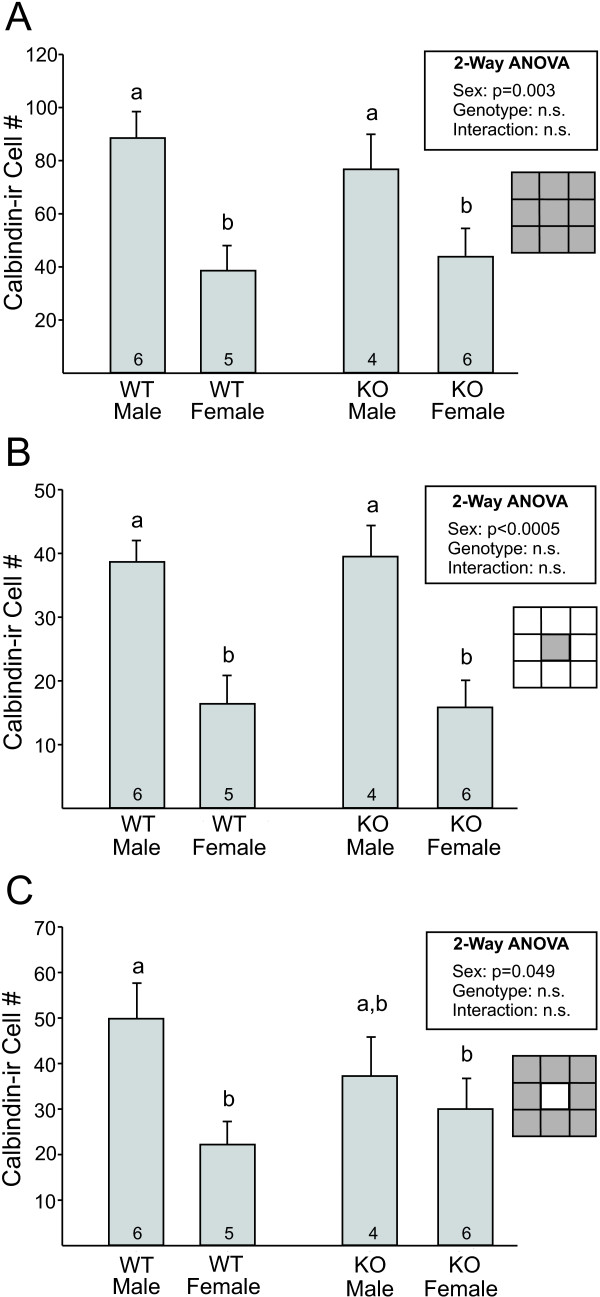
**Grid analysis of calbindin-immunoreactive (ir) cells in and around the calbindin-ir sexually dimorphic nucleus in the preoptic area (CALB-SDN) in wild-type and *Bax *knockout (KO) males and females**. Mean (± SEM) calbindin-ir cell number (A) within the total grid (sum of blocks 1 to 9); (B) in the central block of the grid only (which captures the majority of the CALB-SDN); and (C) in blocks 1 to 4 and 6 to 9 of the grid, reflecting calbindin-ir cells in regions surrounding the CALB-SDN. The number of animals per group is indicated at the base of each bar. Different letters above bars indicate significant differences between groups in *post hoc *tests.

Similarly, a main effect of sex was found for calbindin-ir cell number in grid counts (entire grid: F_1, 17 _= 14.7; *P *= 0.001; central block of the grid: F_1, 17 _= 31.3; *P *< 0.0005; sum of blocks 1 to 4 and 6 to 9: F_1, 17 _= 6.0; *P *= 0.025, Figure 5A-C, respectively). All three analyses yielded no main effect of *Bax *genotype or sex-by-genotype interaction. Although the number of calbindin-ir cells in regions surrounding the cluster appeared lower in *Bax *knockout than in wild-type males (Figure 5C), this was not statistically significant. These findings suggest that *Bax*-dependent cell death does not contribute to cell number in the CALB-SDN or adjacent regions.

#### BNSTp counts

**Figure 6:**
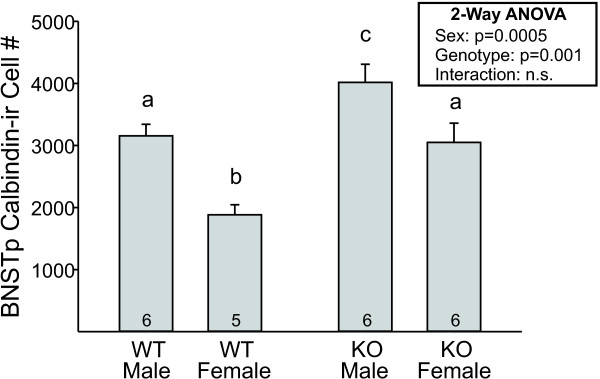
**Mean (± SEM) number of calbindin-immunoreactive (ir) cells in the bed nucleus of the stria terminalis (BNSTp) of wild-type (WT) and *Bax *knockout (KO) males and females**. There was a sex difference as well as a significant effect of *Bax *genotype. The number of animals per group is indicated at the base of each bar. Different letters above bars indicate significant differences between groups in *post hoc *tests.

Calbindin immunoreactivity could be found throughout the BNSTp, although the majority of labeled cells were distributed in the dorsomedial portion of the encapsulated BNSTp and the ventral-most region of the nucleus that dips into the POA. Cell counts revealed a main effect of sex, with more calbindin-ir cells in the BNSTp of males (Figure 6; F_1, 19 _= 19.5; *P *< 0.0005). This is consistent with a previous report that examined calbindin within the encapsulated portion of the BNSTp [29]. In contrast to the CALB-SDN and surround, however, there was also a significant effect of *Bax *gene deletion on calbindin-ir cell number in the BNSTp: *Bax *knockout animals of both sexes had 35% to 40% more calbindin-ir cells than did wild-type mice (F_1, 19 _= 16.0; *P *= 0.001), with no sex by genotype interaction.

### Total cell density in thionin counterstained tissue

The absence of an effect of *Bax *deletion on cell number in the CALB-SDN was somewhat surprising and led us to test for an effect on total cell density in the region. Sections that had been immunoreacted for calbindin were therefore counterstained with thionin, the ellipsoidal contour was positioned as before, and counts of all cells within the contour were made. Calbindin-ir cells made up about 15% of the total cells within the ellipse. Two-way ANOVA on total cell number (calbindin positive plus calbindin negative) revealed a main effect of *Bax *genotype (F_1, 18 _= 5.2; *P *= 0.015), with more cells in *Bax *knockout than in wild-type mice (wild-type male: 786 ± 35, wild-type female: 791 ± 51, *Bax *KO male: 853 ± 32; *Bax *KO female: 938 ± 39). There was no significant effect of sex (F_1, 18 _= 1.3; *P *= 0.27) or sex-by-genotype interaction (F_1, 18 _= 1.01; *P *= 0.33) on this measure.

## Discussion

Male mice have a cluster of calbindin-ir cells in the POA that mirrors a similar cell group in the SDN-POA of rats [28-31]. Although previous studies uniformly report that the volume of this cluster is larger in males, some controversy exists about whether this is due to a sex difference in the number of calbindin-ir cells. Using a uniform contour superimposed in a consistent manner over sections of the POA in males and females, we confirm that the CALB-SDN contains about three times as many cells in male than in female mice. In addition, a single injection of EB on the day of birth completely masculinizes cell number within the CALB-SDN of females.

These findings support and extend the report of Orikasa and Sakuma [31] that male mice have more cells in the CALB-SDN than do females and that five daily injections of EB between postnatal days 1 and 5 masculinized cell number in females. In contrast, cell counts in the CALB-SDN were not affected by adult gonadectomy or hormone replacement [31]. Similarly, in rats, volume of the CALB-SDN was reduced in males by gonadectomy on the day of birth and increased in females treated neonatally with either testosterone proionate or EB [27]. This indicates that sexual differentiation of the CALB-SDN is due to organizational effects of estrogenic steroids. However, there may also be a role for androgens, at least in mice, because disruption of the androgen receptor results in an intermediate phenotype (that is, volume of the CALB-SDN is not significantly different from females or males), as does the neonatal treatment of female mice with the non-aromatizable androgen dihydrotestosterone [28,30].

With the use of grid counts we also examined the number of calbindin-ir cells surrounding the main cluster. Males had more calbindin-ir cells not only within the CALB-SDN, but also in adjacent regions. This supports the interpretation that total calbindin-ir cell number in the POA is sexually dimorphic; we did not find evidence for an equal number of cells that are more scattered in females. A limitation of this conclusion is that our grid counts examined cells distributed in two dimensions (ventral to dorsal and medial to lateral) and only within the confines of the grid. We cannot rule out the possibility that females have more calbindin-ir cells scattered rostrocaudally from the central cluster or beyond the perimeter or our grid, although a simple visual inspection did not suggest either possibility.

The sex difference in cell number in the CALB-SDN mirrors the sex difference in total cell number in the rat SDN-POA which, as described above, is thought to be due primarily to estrogen-regulated developmental cell death [11-13,15,16,19]. We therefore hypothesized that cell death was the mechanism underlying sexual differentiation of the CALB-SDN, and tested this by comparing wild-type and *Bax *knockout mice. Cell number is increased in many brain regions of *Bax *knockouts and several sex differences in the brain and behavior are eliminated in *Bax*-/- mice [25,37-39]. Surprisingly, however, *Bax *gene deletion did not increase the number of calbindin-ir cells in the CALB-SDN or surrounding regions of either sex. Thus, our results do not support a role for cell death in regulating either total cell number or the sex difference in cell number in the CALB-SDN.

The calbindin cluster is often taken as representative of the SDN-POA as a whole. The current findings suggest that either the role of cell death in the SDN-POA should be reconsidered, or that there are different mechanisms underlying the differentiation of calbindin-ir cells and other cells in the nucleus. Several caveats should be considered, however. For example, it is possible that the sex difference in the number of calbindin-ir cells is in fact due to cell death, but it is Bax-independent cell death. This seems somewhat unlikely given the crucial role of Bax and Bcl-2 in controlling cell death throughout the developing brain and the fact that *Bax *deletion increased total cell density in the cluster region. In addition, apoptotic cells, as identified by activated caspase-3 immunoreactivity, are essentially eliminated in the medial preoptic area (as well as most other brain areas) of *Bax *knockout mice postnatally (Ahern T, Carr A and Forger NG, unpublished results), suggesting negligible Bax-independent cell death in this region. If cell death contributes to calbindin-ir cell number in the POA, it would have to be both Bax and caspase-3 independent.

A second possible limitation to the conclusion that cell death does not contribute to development of the CALB-SDN is that presumptive calbindin-ir cells may be rescued from cell death by *Bax *deletion, but fail to make calbindin protein. Supernumerary cells in the BNSTp and anteroventral periventricular nucleus of *Bax *knockout mice express proteins such as vasopressin, NeuN, the androgen receptor (AR) and kisspeptin [36,40,41], indicating that they differentiate normally to the extent of expressing at least some relevant markers. However, the 'extra' cells in *Bax *knockout mice may not always differentiate normally (see, for example, [42]). The inability of cells rescued by *Bax *deletion to specifically express calbindin seems unlikely here, however, because we found a significant increase in the number of calbindin-ir cells in the BNSTp of *Bax *knockout animals.

Taken together, these findings indicate that cell death is not the only mechanism underlying sex differences in cell number in the SDN-POA, at least not the sex difference in calbindin-ir cell number. Differential neurogenesis, migration, or phenotypic differentiation are other possible mechanisms. Previous studies examining the birthdate of cells comprising the SDN-POA of rats have not found evidence for a sex difference in neurogenesis [11,34,43,44]. A sex difference in the migration or aggregation of cells into the nucleus is possible, especially in light of work demonstrating effects of sex and estradiol on cell migration in embryonic slice cultures of the POA [45,46]. We note, however, that if differences in the migration or aggregation of cells account for sexual differentiation of the CALB-SDN, calbindin-ir cells in females must end up quite a distance away from the cluster seen in males (that is, beyond the boundaries of the grid used for counts here). A more likely mechanism may be the differentiation of neurochemical phenotype. That is, more cells may be induced to differentiate into calbindin-expressing cells in males, presumably in response to perinatal exposure to testosterone and its metabolites. Similarly, other sex differences in the medial POA of rats depend on the differentiation of (morphological) phenotype, for example, the morphology of astrocytes is more complex and the density of dendritic spines is greater in the POA of male than in female rats [47,48].

In addition to the sex difference in the CALB-SDN, we also found a sex difference in the number of calbindin-ir cells in the BNSTp. The calbindin-ir cells in the BNSTp have not received much attention although they comprised the largest and most striking accumulation of calbindin-positive cells in our material. In the only other report on these cells that we are aware of, and consistent with the present findings, Büdefeld *et al. *[29] reported more calbindin immunoreactivity in the encapsulated portion of the BNSTp of male than of female mice. This sex difference was seen in animals that had been gonadectomized and treated with testosterone propionate in adulthood indicating that, similar to the sex difference in the CALB-SDN, it is independent of adult hormonal state [29].

Preventing cell death by *Bax *gene deletion increases the number of Nissl-stained cells and AR-expressing cells in the BNSTp and eliminates sex differences in total and AR cell number [25,36]. Deleting *Bax *also increased the number of calbindin-ir cells in the BNSTp here. However, the increase was roughly equivalent (approximately 1,000 cells) in males and females so the sex difference was not eliminated. Thus, although cell death determines the number of potential calbindin-ir cells in both sexes, a second mechanism must be responsible for the sex difference. As for the CALB-SDN, we suggest that this second mechanism is the differentiation of neurochemical phenotype (that is, more cells become calbindin-expressing neurons in males, under the control of hormones or sex chromosomes).

The fact that *Bax *deletion increased calbindin-ir cell number in the BNSTp but not the CALB-SDN raises the question of what underlies this regional difference. Although calbindin has been used as a 'marker' of the SDN-POA it is also a calcium binding protein associated with neuroprotection (see, for example, [49-51]). In rats, calbindin is expressed at higher levels in males in the preoptic area/mediobasal hypothalamus generally, and the SDN-POA specifically, prior to the period of sexually dimorphic cell death [27,52,53]. It is therefore possible that perinatal testosterone directs more cells to become calbindin-expressing in males and that those cells with calbindin protein are relatively protected from cell death. That is, *Bax *gene deletion may have had no effect on the number of cells in the CALB-SDN because calbindin-expressing cells are normally spared from pruning in wild-type animals. To our knowledge, it is not known if the ontogeny of calbindin expression differs between the CALB-SDN and BNSTp, but an earlier emergence of calbindin in the CALB-SDN could be one possible explanation for why *Bax *deletion influences calbindin cell number differently in the two nuclei.

## Conclusions

Male mice have more cells in the CALB-SDN and surrounding areas than do females, and these sex differences are prevented by treating females with estradiol at birth. Although the sex difference in the CALB-SDN is due to cell number, it does not appear to depend on developmental cell death. This suggests that cell death is not the only mechanism controlling sex differences in the SDN-POA and caution may be warranted in using findings from the subpopulation of cells expressing calbindin as a proxy for processes operating in the SDN-POA as a whole.

## Competing interests

The authors declare that they have no competing interests.

## Authors' contributions

RFG participated in study design, collected and sectioned brains, performed immunocytochemistry, analyzed data, and helped draft the manuscript; MMV treated neonatal mice and collected and sectioned brains; NGF conceived of the study, participated in its design, and drafted the manuscript. All authors read and approved the final manuscript.
